# Novel ophthalmic hyaluronic acid-hydrogel with curcumin nanoparticles for enhanced healing of ulcerative keratitis in rabbit model

**DOI:** 10.1038/s41598-024-74195-w

**Published:** 2024-10-04

**Authors:** Zahra Sohani, Shahram Jamshidi, Mohammad Kazem Koohi, Javad Malakootikhah, Mohammad Abarkar, Diba Golchin, Sarina Roshani, Haniyeh Naghdi, Negin Aghajanpour-moghaddam-gazafroudi, Gazafroudi, Nava Amjadi, Reihaneh Izadi-niaki

**Affiliations:** 1 Faculty of Veterinary Medicine, University of Tehran, Tehran, Iran; 2 Department of Internal Medicine, Faculty of Veterinary Medicine, University of Tehran, Tehran, Iran; 3 Department of Comparative Biosciences, Faculty of Veterinary Medicine, University of Tehran, Tehran, Iran; 4 Department of Nanobiotechnology, College of Interdisciplinary Science and Technologies, University of Tehran, Tehran, Iran; 5 Department of Clinical Science, Faculty of Veterinary Medicine, Karaj Branch, Islamic Azad University, Karaj, Iran; 6 Department of Pathology, Faculty of Veterinary Medicine, University of Tehran, Tehran, Iran; 7 Department of Surgery and Radiology, Faculty of Veterinary Medicine, University of Tehran, Tehran, Iran

**Keywords:** Curcumin nanoparticles, Hyaluronic acid, Β- cyclodextrin, Hydrogel, Ulcerative keratitis, Pharmaceutics, Drug development, Drug discovery, Medical research, Diseases, Eye diseases, Corneal diseases

## Abstract

Corneal ulcers, whether melting or indolent, are common in humans and companion animals. Treatment involves local administration of antibiotic eye drops and corneal healing drugs. Compared to traditional treatments for ulcerative keratitis, herbal medicines offer unique advantages, such as potent anti-inflammatory effects and inhibition of proinflammatory cytokines. Curcumin, extracted from the Curcuma Longa plant, possesses extensive pharmacological properties, such as anti-inflammatory, anti-cancer, and antioxidant properties, and is used in various medicines. In this study, we developed a novel ophthalmic drop hydrogel using a formulation of Curcumin NPs encapsulated with β-cyclodextrin and hyaluronic acid, to accelerate corneal healing and improve the quality of healed structures. The formation of Curcumin NPs into Hyaluronic acid-based hydrogels was characterized by zeta, FTIR, and scanning electron microscope (SEM) analyses. A total of 25 healthy male New Zealand Albino rabbits were experimentally induced with ulcerative keratitis and treated individually with topical medication. Rabbits were divided into five groups. Fluorescein dye staining, corneal clarity score, Schirmer tear test, proinflammatory cytokine measurement, and pathologic factors assessments were used to evaluate the optimised Curcumin NPs with β-cyclodextrin in Hyaluronic acid hydrogel. Our results demonstrated that the optimized Curcumin NPs with β-cyclodextrin in hyaluronic acid hydrogel significantly reduced the frequency of medication administration compared to conventional therapies, enhancing the quality of healed structures and effectively treating ulcerative keratitis. All findings in this study provide new insight into designing and fabricating novel ophthalmic medicine for ulcerative keratitis for topical usage.

**Supplementary Information:**

The online version contains supplementary material available at 10.1038/s41598-024-74195-w.

## Introduction

The cornea is a transparent, non-vascular, non-pigmented, and non-keratinized tissue in the eye’s outermost layer^1^. Corneal ulcers, also known as loss of surface epithelial cell barrier, are among the most common ocular diseases diagnosed in companion animals and people; associated with loss of corneal layers may result from many factors, such as foreign body trauma, laceration, keratoconjunctivitis sicca, trichiasis, ectropion, lagophthalmos, heat, smoke, or alkali burn^2–5^.

Corneal ulcers are a major cause of blindness worldwide^6^. Postponed management of corneal ulcers leads to various complications such as glaucoma, endophthalmitis, blepharospasm, hyper-lacrimation, and aqueous flare^7^. Moreover, they can affect the smoothness and curvature of the cornea, leading to inappropriate focusing of images on the retina^8^. Corneal ulcers are also susceptible to secondary microbial infections caused by microorganisms, such as bacteria and fungi, which can release proteolytic enzymes and toxins, further complicating the healing process. Therefore, accelerating the healing of corneal ulcers is crucial to maintain ocular health^9^. The corneal recuperation process is a complex interaction of cytokines, growth factors, and proteinases discharged from harmed epithelial cells, inflammatory cells, and activated stromal fibroblast cells, which stimulates proliferation, migration, differentiation, and adhesion of cells that participate in wound healing^10,11^. Traditional strategies based on N-acetylcysteine in the treatment of corneal oxidative disorders, such as after alkali-burned ulcerative keratitis, are limited owing to their high cost, high toxicity, and low efficiency^12^.

Curcumin (1,7-bis[4-hydroxy-3-methoxyphenyl]-1,6-heptadiene-3,5-dione), an FDA-approved yellow-colored polyphenol extracted from the Curcuma longa plant, is a potential herbal medicine with extensive pharmacological properties, such as anti-inflammatory, anti-cancer, anti-oxidant, anti-angiogenic, antimicrobial, and wound healing effects^13–15^. The anti-inflammatory action of curcumin has been demonstrated in various systemic inflammatory pathological pathways^16^. Curcumin diminishes proinflammatory cytokines by inhibiting the IκB kinase, nuclear factor kappa B (NF-κB), and mitogen-activated protein kinase (MAPK) signalling pathways. It also downregulates the expression of 5-lipoxygenase, cyclooxygenase-2, and nitric oxide synthase^17–20^. Recent studies have demonstrated the potential therapeutic activities of curcumin in the treatment of ophthalmological diseases, including keratoconjunctivitis sicca, glaucoma, uveitis, age-related macular degeneration, and diabetic retinopathy^21–27^.

Hyaluronic acid is a linear, unbranched polymer connective tissue polysaccharide that promotes corneal healing by stimulating migration during re-epithelialization, mucus adhesion, and proliferation of corneal epithelial cells. Additionally, it possesses anti-inflammatory and antioxidant properties. Hydrogels made from hyaluronic acid exhibit wound healing properties, facilitate cell conveyance, assist in local drug delivery, decrease drug susceptibility to enzymatic degradation, and decrease the number of applications of topical formulations inside the eye daily^28,29^.

Numerous formulations based on encapsulation in polymer nanoparticles, nanogels, surfactants, proteins, bilayers, phospholipids, and conjugates have an approach to improve low solubility in aqueous solutions (≈ 20 µg/mL), poor bioavailability at physiological pH, destitute absorption, rapid metabolism, and elimination of curcumin^30–34^. Among the carriers for the encapsulation of curcumin, β-cyclodextrin, a semi-natural, nontoxic oligosaccharide composed of seven α-(1,4)-linked glucopyranose subunits, can stabilize drugs, reduce irritation, and enhance drug solubility and permeability^20,35^. Hydrogels are highly hydrated materials with three-dimensional (3D) network structures formed by crosslinking hydrophilic polymers. These materials can contain large quantities of water. In addition, polymeric nanoparticles based on hydrogels have improved the adherence of pharmaceutical ingredients for wound repair. They also help maintain viable medication levels over extended periods and allow for less frequent dosing^36–38^.

N-acetylcysteine, an acetylated form of the amino acid l-cysteine, has antioxidant properties by reducing the expression of the NF-κB transcription factor and mucolytic and anti-collagenolytic activities when applied topically in corneal ulcerations in animals and humans^39–41^.

In the current study, aiming at efficient therapy of ulcerative keratitis, we developed topical eye drops based on Curcumin NPs encapsulated with beta-cyclodextrin stabilised in Hyaluronic acid hydrogel. We compared it with a topical eye drop of N-acetylcysteine 5% in rabbit eyes.

## Experimental & methods

### Materials

All chemicals used in the experiments were analytical and utilized without additional treatment. Curcumin NPs CoruMax and Hyaluronic acid were purchased from Nanoage Co. (Tehran, Iran). Artificial tears (Sina Darou, Iran) were used to prepare all aqueous solutions using a Millipore system under sterile conditions. (18.2 MΩ; Millipore Co., USA). Lecithin and NaOH 1 M were acquired from Merck (Darmstadt, Germany).

### Synthesis of curcumin NPs into hyaluronic acid-based hydrogel

In a separate container, 0.02 g of curcumin NPs encapsulated with β-cyclodextrin were dissolved in 4 ml of methanol. The curcumin nanoparticles were prepared using β-cyclodextrin as the encapsulating agent, which acts as a polymeric host, facilitating the inclusion of the hydrophobic curcumin within its hydrophilic cavity. This encapsulation process not only enhances the stability of curcumin but also ensures its controlled release within the hyaluronic acid-based hydrogel matrix^42^. The two resulting solutions were combined and rotated for an hour at a temperature of 60 ºC with a speed of 240 rpm and under vacuum conditions. Subsequently, 8 ml of distilled water and 0.02 g of Hyaluronic acid were added to the hydrolysis process. Then, it was rotated for 30 min at a speed of 240 rpm without a vacuum. The resulting solution was stirred for 20 min at a speed of 800 rpm at a temperature of 60 ºC. Finally, it underwent three cycles of 3 min with a rest period of 1 min. The resulting product was then freeze-dried^43^.

### Characterization

The Curcumin NPs into Hyaluronic acid-based hydrogel was dispersed in ethanol before being deposited onto a carbon-coated copper grid. Scanning electron microscopy (SEM) was performed using a Nova Nano SEM 450 scanning electron microscope operated at 5 kV to investigate the morphological and structural features of the Curcumin NPs encapsulated within the Hyaluronic acid-based hydrogel. Specifically, SEM was employed to observe the size, shape, surface characteristics, and potential aggregation of the nanoparticles within the hydrogel matrix. The particle size, polydispersity index (PDI), and zeta potentials of synthesized samples were evaluated using a dynamic light scattering (DLS) device (Zetasizer Nano ZS90, Malvern, Worcestershire, UK). Fourier transform infrared spectrometric (FTIR, Shimadzu 8400 S, Japan) analysis was applied to identify the chemical structure of the Curcumin-loaded hydrogel.

### Animals & experimental design

The experimental phase was conducted on 25 male New Zealand Albino rabbits aged 3–4 months and weighing 3 to 3.5 kg obtained from the University of Tehran Research Council, Tehran, Iran. They were kept in individual cages with noise of less than 60 dB. Throughout the study, rabbits consumed a standard commercial pellet diet and free water ad libitum. All animal studies, including experimental procedures and euthanasia, were approved by the Iranian Animal Ethics Committee (protocol number IR. UT. VETMED. REC.1402.017) and performed under the supervision of Iran SPCA, following the animal research: Reporting of In Vivo Experiments (ARRIVE) guidelines. The entire methods were performed by the relevant guidelines and regulations. After the study all rabbits were humanely euthanized. The euthanasia process was carried out following the American Veterinary Medical Association (AVMA) Guidelines for the Euthanasia of Animals. Specifically, the rabbits were first anesthetized using an intraperitoneal injection of ketamine (35 mg/kg) and xylazine (5 mg/kg) to ensure they were anesthetized. Following confirmation of anesthesia, euthanasia was performed using an intravenous overdose of pentobarbital sodium (150 mg/kg).

Before the study, ophthalmic examination, including slit lamp biomicroscopy (SL-17; Kowa Company, Ltd., Tokyo, Japan), indirect ophthalmoscopy (Keeler Vantage; Keeler Instruments, Inc., Broomall, PA, USA), rebound tonometry (TonoVet; Icare FinlandOy, Espoo, Finland), fluorescein staining (Kashurg, India) and Schirmer tear test (KSIPL, India) of both eyes were performed. All animals displayed good health and were free of clinically observable ocular abnormalities.

### Alkali burn-induced corneal ulceration

The eyes were treated preoperatively with chloramphenicol 0.5% ophthalmic drops (Sina Darou, Iran) four times a day for one day before the surgery to prevent unintended bacterial infection. Rabbits were anaesthetized via intramuscular administration of xylazine 2% (5 mg/kg, Alfasan, Netherlands) and ketamine (35 mg/kg, Bremer Pharma, Germany) and followed by topical application of tetracaine 0.5% eye drops. (Anestocaine, Sina-Daru, Iran).

**Fig. 1:**
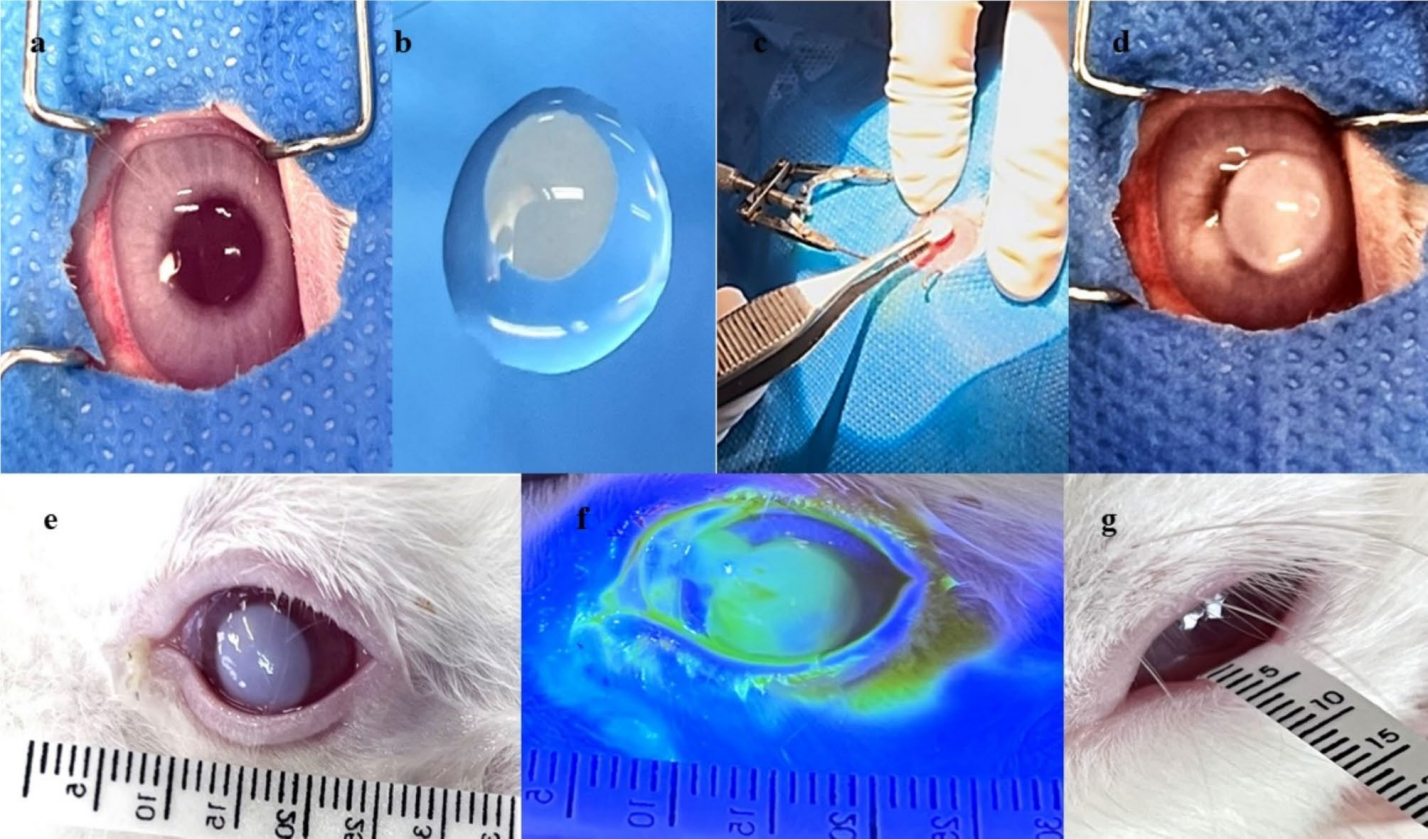
Experimental alkali burn induction in rabbits’ eyes. (**a**) Pre-surgery eye preparation (**b**) filter paper soaking in NaOH 1M (**c**) Ulcer induction proceeding (**d**) Ulcer appearance post-surgery (**e**) Ulcerative keratitis (**f**) Ulcerative keratitis confirmation (**g**) Post-surgery Schirmer tear test.

After aseptic animal preparation, a 7 mm diameter circular section of filter paper, soaked in 1 M NaOH solution for 30 s through sterilization, was applied to the central region of the cornea of the right eye for 30 s. The cornea was flushed with a continuous flow of 2 ml sterile normal saline 0.9% for 1 min. One drop of chloramphenicol 0.5% (Chlobiotic, Sina Darou, Iran) was used immediately to avoid infection^44–47^. The same veterinary surgeon performed all the alkali burns under a surgical microscope to minimize variability. Positive fluorescein staining confirmed the properties of the burnt ulcers. The alkali burn induction process is shown in Fig. 1. The rabbits were divided into five groups at random, with five animals in each.

- Group l: The untreated group received artificial tear (25 µl, BID, Sina Darou, Iran).
- Group II: treated with Curcumin NPs into Hyaluronic acid-based hydrogel topical ophthalmic drop (25 µl, diluted in artificial tear, BID).
- Group III: treated with Curcumin NPs topical ophthalmic drop (25 µl, BID).
- Group IV: treated with Hyaluronic acid hydrogel 2% topical ophthalmic drop (25 µl, BID).
- Group V: treated with NAC 5% topical ophthalmic drop (25 µl, TID, Exir darou, Iran).

For ocular topical treatment, the drugs in groups I, II, III, and IV were applied ocular topically twice daily with 25 µl volume per eye and in group V three times a day with 25 µl volume per eye for seven days^48,49^.

The rabbits in each group with injured eyes were treated with chloramphenicol (1 drop, once a day) to prevent secondary bacterial infection.

**Fig. 2:**
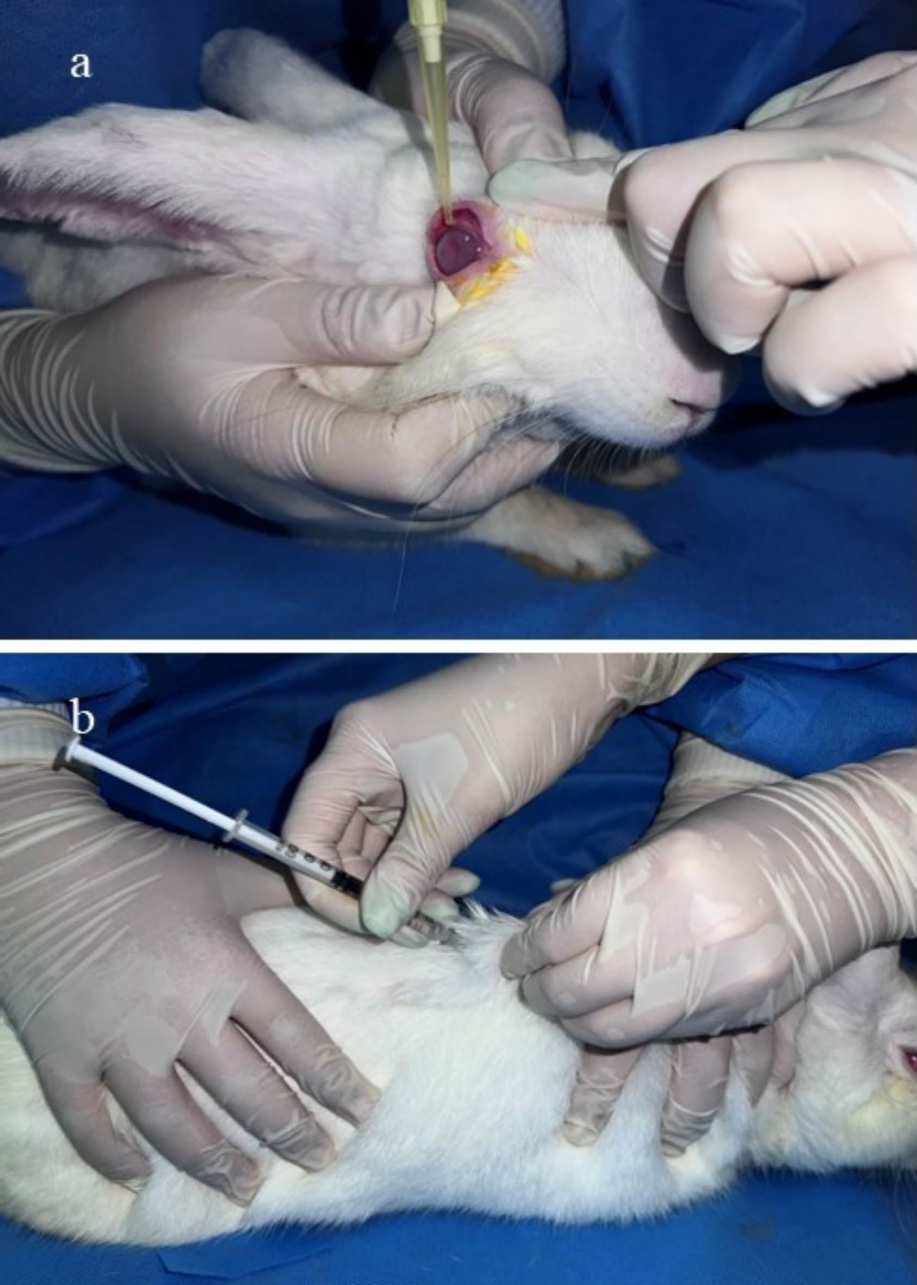
(**a**) Administrating ophthalmic drops to a rabbit’s eye (**b**) Administrating tramadol subcutaneously.

For the preparation of eye drops, all of the solutions used in this study were dilated in artificial tears (Sina Darou, Iran) under sterile conditions, and all animals received tramadol injections due to pain relief (5 mg/kg, SC, once a day, Darou Pakhsh, Iran). The method of administrating medications is shown in the Fig. 2.

### Neuro-ophthalmic examination

Neurological assessments of the rabbits were performed in a dark room with magnification, and focal light sources were used daily. These assessments included the menace response, palpebral, pupillary light, and dazzle reflexes. Ophthalmic examinations included indirect ophthalmoscopy, the Schirmer’s tear test, and fluorescein dye staining.

#### Indirect ophthalmoscope examination

Re-examinations were scheduled on the 1st, 3rd,5th, and 7th post-surgery days. Corneal clarity score was evaluated with an indirect ophthalmoscope (0–4 stages), G0: No fundus reflection is visible on retro illumination, G1: A fundus reflection is visible with retro illumination as described in G0, G2: A 0.1-mm-diameter light beam from a handheld slit lamp is visible on the anterior side of the iris and lens with the naked eye; G3: Gross fundic features are visual when viewed by indirect ophthalmoscopy, although fine details are not precise, and G4: Fine details of the fundic features are visible with indirect ophthalmoscopy as described in G3^5^.

#### Schirmer tear test

Schirmer tear tests (STT) were performed on the 2nd, 4th, 6th, and 8th postoperative days after surgery.

The folded head of the Schirmer tear test strips was placed in the middle to the temporal third of the lower eyelid for 60 s^50^.

#### Fluorescein dye staining

The orange dye fluorescein strips were scheduled on the 1st, 3rd, 5th, and 7th post-surgery days and set lightly to the conjunctival surface for one second^50^. The eyes were then monitored with blue light from cobalt using a direct ophthalmoscope. The total wound area in square millimeters, colored greenish, was determined using the image processing program GIMP version 2.8.14 (provided in the public domain by https://gimp.org) and calculated from $\:total\:wound\:aea=\:\frac{number\:of\:woun{d}^{{\prime\:}}s\:pixels}{pixel\:\left(mm2\right)}$. A pixel’s whole wound is defined as the total number of pixels representing the wound area, and a pixel (mm^2^) is defined as the number of pixels in one square millimeter^51^.

### Cytokine measuring

Samples of aqueous humor were collected under sterile conditions prior to euthanasia. On the 8th day, limbal paracentesis of approximately 100 to 200 µl of aqueous humor was performed in the peripheral cornea next to the limbus using a 27-gauge needle of the anterior chamber without contact with intraocular structures. Each sample of aqueous humor was centrifuged for 5 min at 3000 g revolutions per minute (rpm) to separate cells from the fluid. Fifty microliters of supernatant were transferred into a sterile microfuge tube and stored at − 80 °C until cytokine levels were assessed.

IL6 and TNF-α concentrations were measured using ELISA according to the manufacturer’s instructions (Wuhan Boster Biological Technology, China), with a 1–1000 pg/ml detection range.

### Histopathological examination

The eyes were fixed in a 10% neutral buffered formalin solution for 24 h and sectioned at 4–6 μm thickness. The sections were stained with Hematoxylin and Eosin (H&E) and examined under light microscopy (Olympus, Japan)^52,53^.

### Statistical analysis

Descriptive data are presented as mean ± SEM. Statistical analyses were performed using SPSS version 27.0. The Kolmogorov-Smirnov test was used to check the normality of the distribution. Statistical analysis between the five groups for STT and fluorescein staining was performed using the Friedman test, and for CCS, it was done using the Kruskal–Wallis test. In the histopathological evaluation, a score (1) was assigned to the positive ranks and a score (0) to the negative ranks. The algebraic sum of the scores obtained from these ranks was considered a general index of histopathology, which was analyzed using the Kruskal–Wallis test^54^. The thicknesses of the corneal layers were statistically analyzed using the One-Way Analysis of Variance (ANOVA) test. ANOVA was used to compare the proportions of cytokine measurements. Mann–Whitney U test compared each group and the control–Whitney U test pairwise. Statistical significance was set at 0.05 significant.

### 10 publicity available data

The data that support the findings of this study are openly available in an Excel file in the supplementary data.

## Results and discussion

### Synthesis and structural characterization

**Fig. 3:**
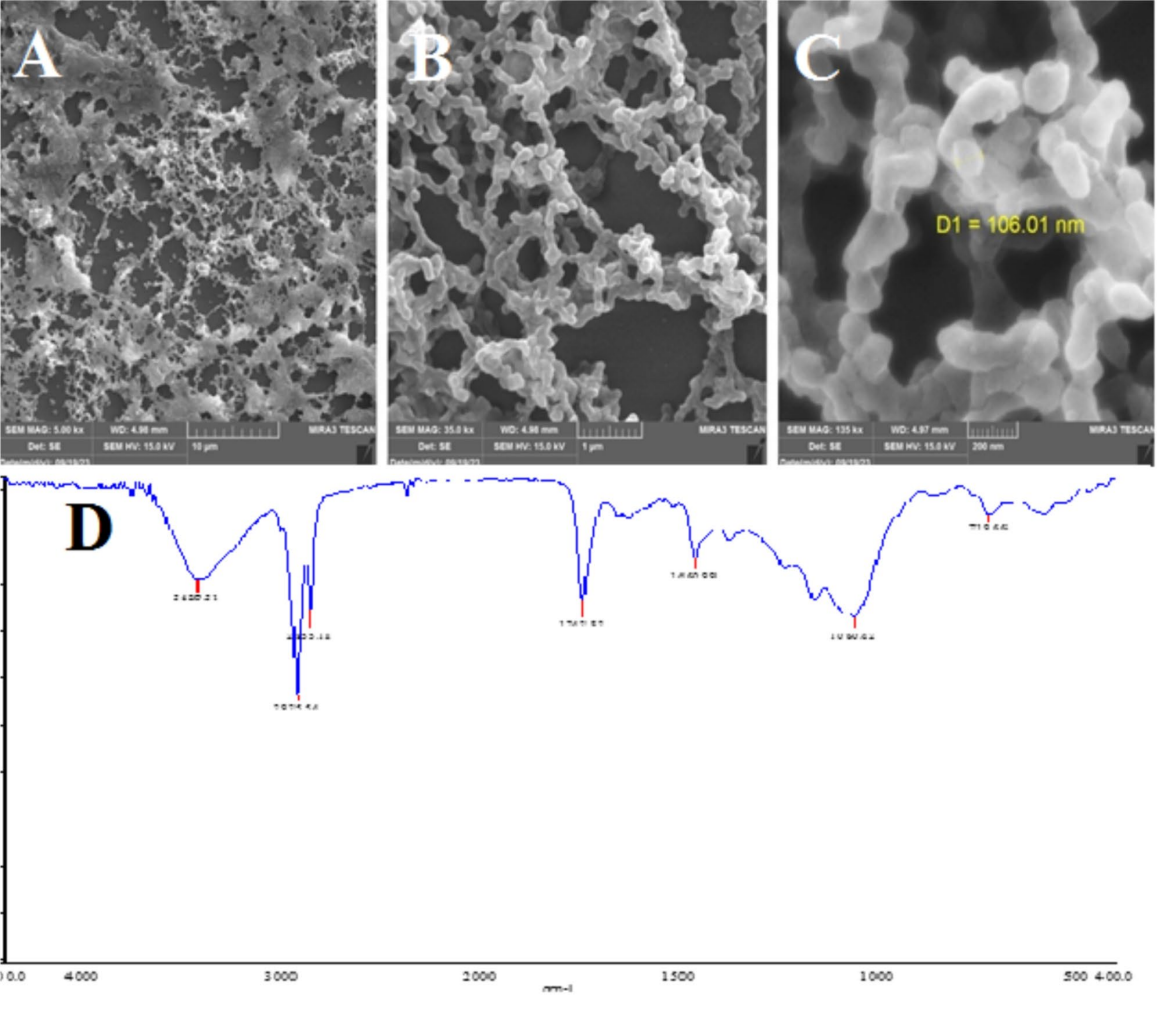
SEM images of Curcumin NPs into Hyaluronic acid-based hydrogel at the scale of (**A**) 10 μm, (**B**) 1 μm and (**C**) 200 nm. (**D**) FTIR analysis hyaluronic acid-curcumin sample at spectrum of 400 to 4000 cm^–1^.

SEM images were used to investigate the morphological and structural features of Curcumin NPs into Hyaluronic acid-based hydrogel, and zeta analysis was used to analyze the structure and chemical modification of Curcumin NPs into the Hyaluronic acid-based hydrogel. SEM images of the Hyaluronic acid-curcumin sample revealed a well-defined network of nanoparticles, predominantly exhibiting smooth spherical and rod-shaped morphologies. Overlapping of these nanoparticles within the hydrogel matrix led to the observation of additional complex morphologies. The SEM analysis also confirmed the size distribution of these nanoparticles, which was approximately 106 nm, consistent with the size expected from the synthesis process. The morphological structure and size distribution of different Curcumin NPs into Hyaluronic acid-based hydrogel were determined and are shown in Fig. 3, according to the results from SEM images by loading drugs. SEM analysis indicated that the average size of the nanoparticles was approximately 106 nm. This size is notably smaller than the size observed through zeta analysis, which measured approximately 440 nm with a PDI of 0.8, suggesting the presence of some aggregation or larger clusters in the solution phase. The surface charge of -19 mV, as measured by zeta analysis, supports the stability of the nanoparticles, with minimal accumulation observed. Based on the results of the analysis center, the curcumin NP-HA nano-powder sample was polydisperse with no observed accumulation. With a PDI value of 0.8, the nanoparticles were estimated to be approximately 440 nm in size, which was expected because the curcumin NPs were coated with hyaluronic acid. The PDI value of 0.8 demonstrates the existence of huge particles or aggregates in the NP-HA nano-powder sample with inhomogeneity.

FT-IR analysis of the hyaluronic acid-curcumin sample confirmed the successful encapsulation of curcumin within the β-cyclodextrin polymer, as evidenced by the characteristic peaks of cyclodextrin in the spectrum. The hydroxyl groups of curcumin were directly coupled to the carboxylic groups of HA, facilitated by the β-cyclodextrin, which acted as a polymeric host during the nanoparticle formation. The broad band around 3400 cm^-1^, associated with O-H stretching, and the peaks around 1740 cm⁻¹ and 1600 cm⁻¹, related to the C = O stretching, further confirm the interaction between curcumin, β-cyclodextrin, and hyaluronic acid. The formation of the HA-Cur structure was also confirmed by a broad band around 3400 cm^-1^, which was assigned to O-H groups in HA. A relatively sharp peak at 2930 Cm^-1^ was related to the phenolic OH of curcumin in the hydrogel structure. The peaks around 1740 Cm^-1^ and 1600 Cm^-1^ were assigned to the C-O stretching frequency of curcumin’s dichtonic function. By comparing the obtained data with the presented analysis in the previous studies, the formation of the NP-HA nano-powder can be confirmed.

### Neuro-ophthalmic results

In this study, we verified the efficacy of Curcumin NPs into Hyaluronic acid-based hydrogel as an herbal anti-inflammatory agent that plays a vital role in corneal wound healing. By examining the changes in fluorescein dye staining, Schirmer tear test, corneal clarity score, histopathologic factors, and cytokine measurements, we aimed to gain insights into the potential consequences of Curcumin NPs into Hyaluronic acid-based hydrogel on corneal ulcers and provide a foundation for future research in this area. Treatment of ulcerative keratitis depends on the severity, presence of infection, and development of corneal sequestrum. Broad-spectrum topical antibiotics and medications that prevent the breakdown of collagen, such as N-acetylcysteine, are recommended for treatment. Topical corticosteroids, as anti-inflammatory agents, are contraindicated for corneal ulcers because they can delay corneal healing, increase the risk of corneal melting caused by destructive enzymes produced by bacteria and inflammatory cells, increase intraocular pressure and cataracts, and suppress the host response, thereby increasing the risk of ocular infection^55^. Only 20% of the applied dose reaches the cornea due to the blink reflex, high tear turnover rate, and lacrimal drainage, which leads to low bioavailability of topical drugs^56,57^. Owing to its low bioavailability, frequent topical administration during the long course of the disease may lead to various complications, such as dry eye, chronic allergy, and intraocular hypertension, among others^38^. Novel drug delivery systems, such as hydrogels, are being developed to overcome the low bioavailability of drugs and deliver sufficient medical agents to the cornea. They provide sustained drug release, increase retention time, and can respond to stimuli, making them a suitable option for corneal drug delivery^57^.

Chemical injuries to the anterior portion of the eye caused by alkali burns are the most serious. (1 mm) Alkalis increases tissue pH levels and causes saponification of fatty acids in cell membranes, ultimately resulting in cellular destruction and further tissue damage and inflammation^58^.

**Fig. 4:**
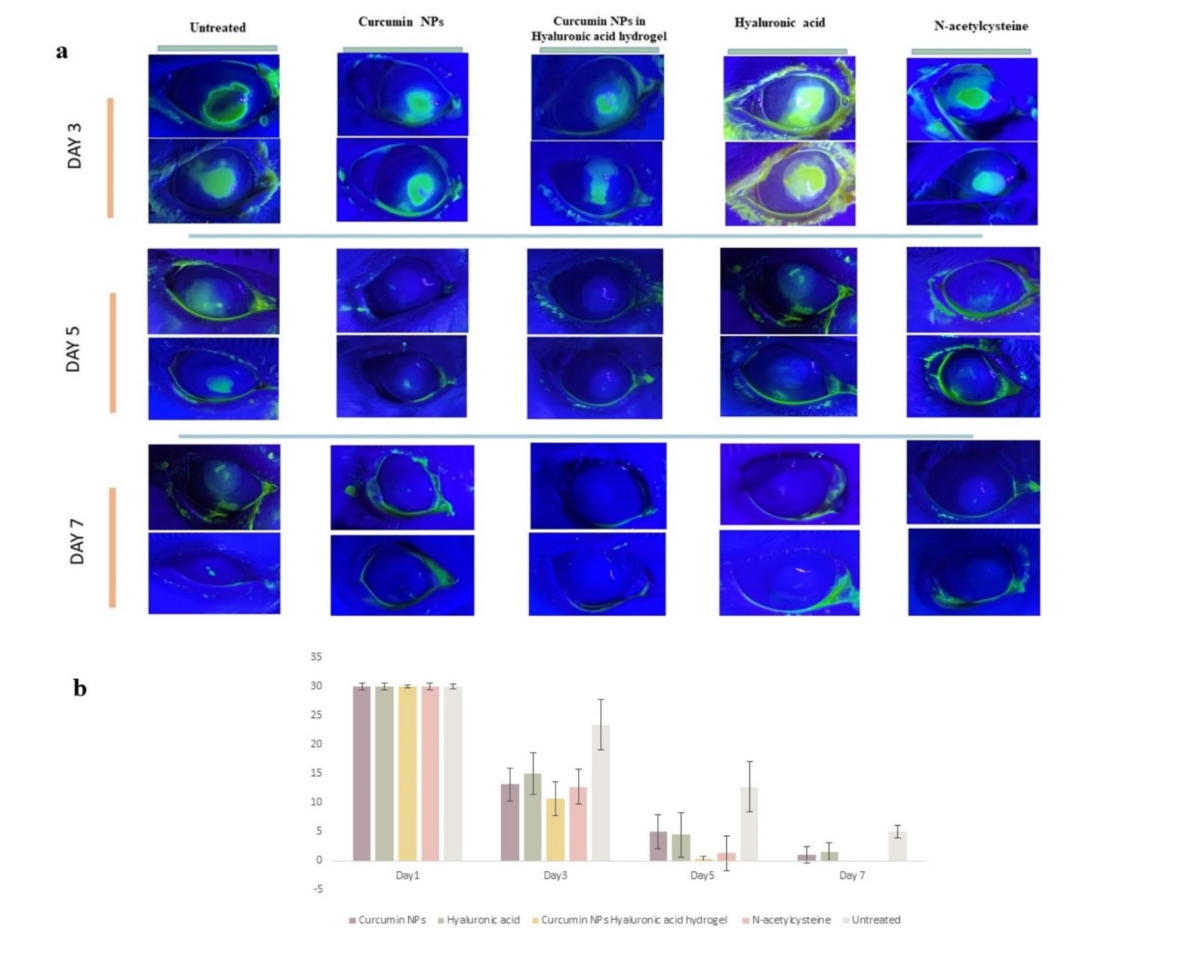
(**a**) Reduction of the corneal damage in the rabbits’ eyes in fluorescein dye staining (**b**) Comparison the corneal ulcer sizes between groups.

As shown in Fig. 4, the corneal epithelial healing rate in the Curcumin NPs into Hyaluronic acid-based hydrogel group was significantly different from the control group 3 days after alkali burn induction (*p* < 0.05)The corneal epithelium defect in the treated group rabbits was significantly decreased compared to the defect size in the untreated group on days 3,5 and 7. Moreover, Curcumin NPs into Hyaluronic acid-based hydrogel group showed significantly different and better corneal epithelial healing. Fluorescein is retained by hydrophilic structures such as corneal stroma. Within minutes of injury, epithelial cells surrounding the margin of the wound begin to slide and cover the affected area. Mitosis occurs once the defect is covered and the multilayered epithelial surface is gradually reconstituted^50^.

Previous research has demonstrated the efficacy of N-acetylcysteine in reducing the size of corneal ulcers^59^. We observed significant differences in ulcer size reduction and physical wound closure in all groups throughout the examination period. (*p* < 0.01) Previous studies indicate that treating corneal injuries involves not only the closure of physical wounds but, more importantly, restoring orderly tissue arrangement, clarity, and vision function^3^.

**Fig. 5:**
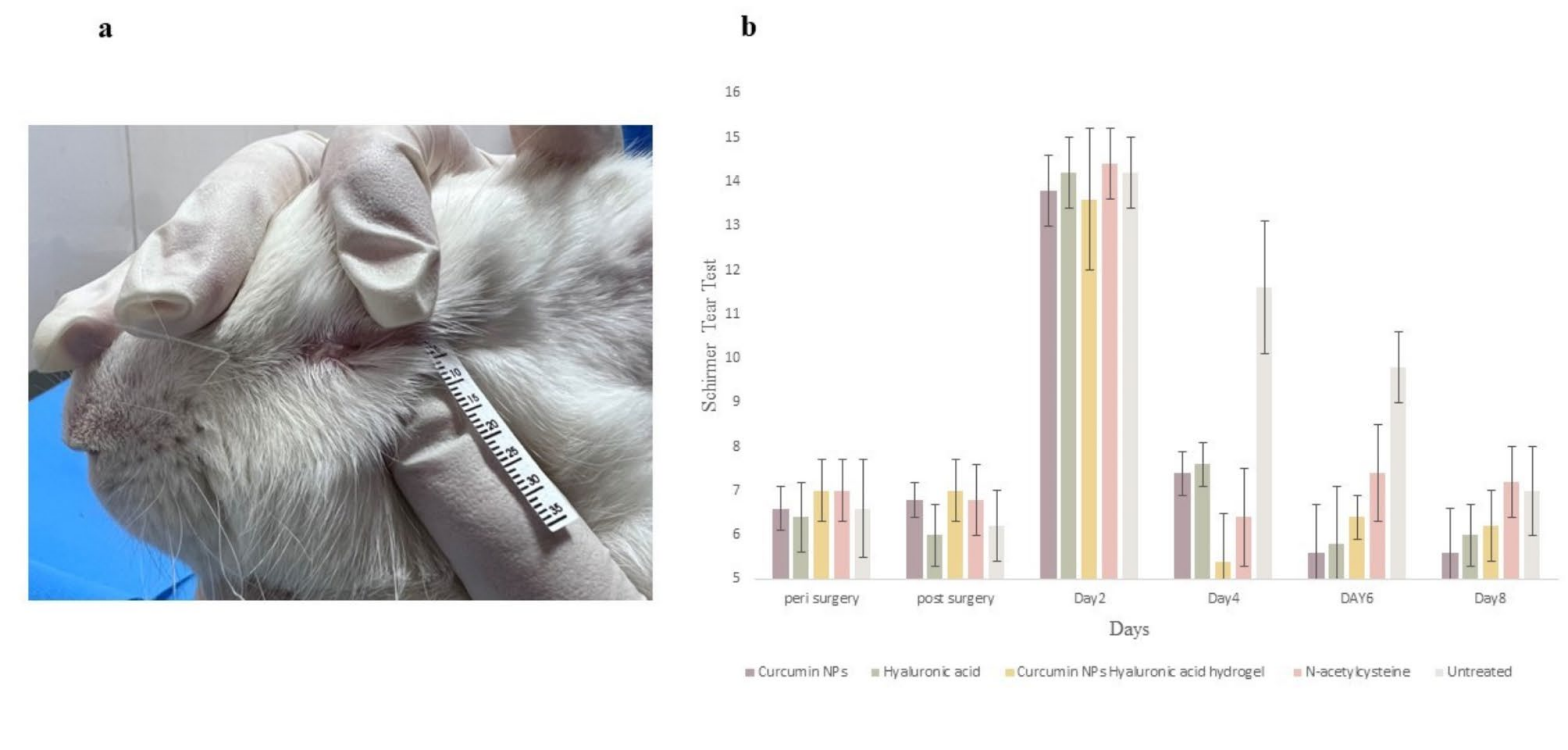
(**a**) Applying a Schirmer tear test strip into the rabbit’s eye (**b**) Comparison of the tear production between groups.

**Fig. 6:**
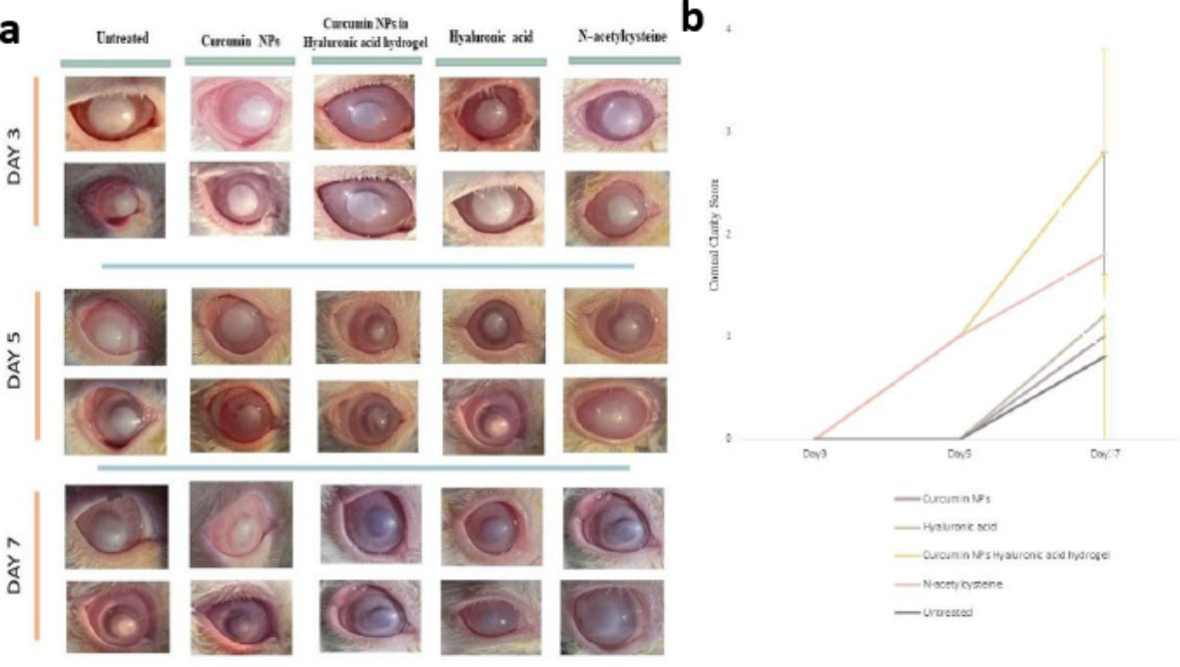
(**a**) Corneal cloudiness and clarity (**b**) Comparison of the corneal clarity scores between groups.

As depicted in Fig. 5, hyper-lacrimation in the injured eye of the treated group increased significantly on day two and returned to normal on day four. Conversely, hyperlacrimation persisted in the untreated group until day six (*p* < 0.01). The Schirmer tear test measures basal and reflex production of the aqueous segment of the precorneal tear film that can determine hyper-lacrimation due to eye injury. A significant difference in corneal clarity was observed on day 7 with Curcumin NPs into Hyaluronic acid-based hydrogel treatment compared to the other groups, as shown in Fig. 6 (*p* < 0.01). During the observation using the indirect ophthalmoscope, the cloudy cornea lasted until the end of the study. The cornea remains transparent owing to the highly regular arrangement of the stromal collagen fibrils. Corneal opacification results from decreased corneal clarity, which leads to a cloudy or hazy appearance. This may be caused by corneal edema, epithelial ulceration, keratinisation, or corneal neovascularization^50^.

These results demonstrated that treating Curcumin NPs into Hyaluronic acid-based hydrogel could restore corneal transparency and reduce ulcer size when applied to alkali burn-induced ulcerative keratitis.

### Pathology results

**Fig. 7:**
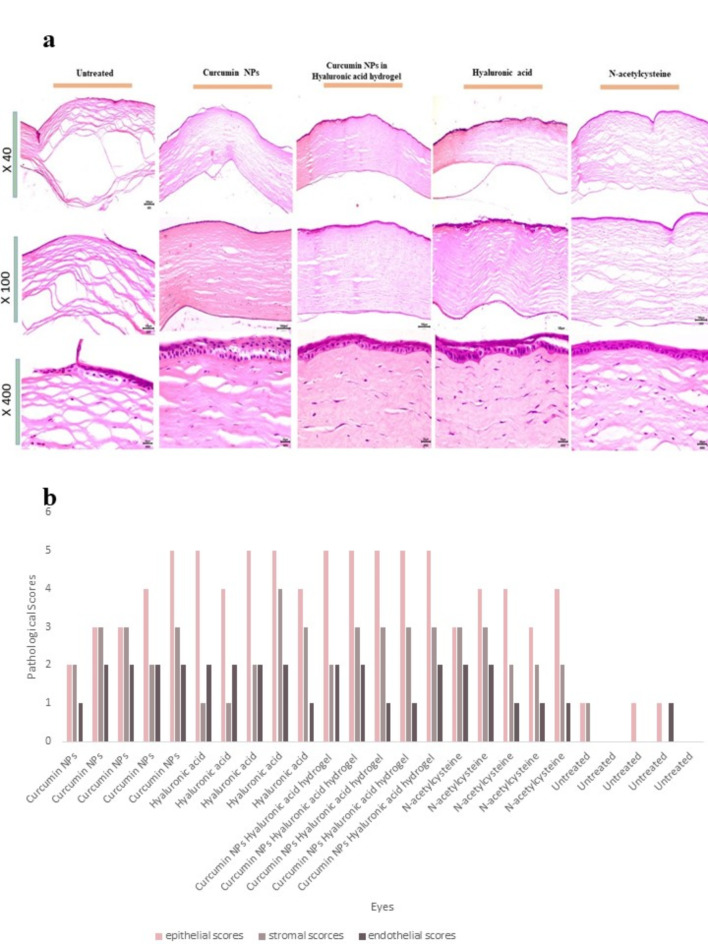
(**a**) H&E staining of the eyes (**b**) Comparison of the pathological factors between groups.

Curcumin NPs into Hyaluronic acid-based hydrogel-treated and hyaluronic acid-treated rabbits significantly increased the total epithelial thickness compared to that in the non-treated group, indicating enhanced epithelial remodeling and recovery. Hyaluronic acid promotes corneal epithelial wound healing by stimulating the migration, adhesion, and proliferation of the corneal epithelium, increasing the remodeling of the extracellular matrix, organizing collagen deposition sites, reducing the expression of fibrotic markers and antioxidant activity, and activating CD44. Moreover, Hyaluronic acid prevents postoperative fibrosis^29,60^. Other researchers have verified the efficiency of crosslinking Hyaluronic acid hydrogel for ophthalmic use and corneal ulcers. Many studies in companion animals and humans have confirmed the safety of Hyaluronic acid in the ocular tissues^28,61,62^. Cross-linked Hyaluronic acid polymer matrix enhances ocular drug bioavailability by spreading hyaluronic acid molecules over the cornea during blinking and slowing down hyaluronidase enzymatic invasion^61,63,64^. After one week, corneal recovery scores were compared across different treatment groups, including epithelial, stromal, and endothelial healing. The epithelial scores included observations of various characteristics such as regrowth or focal loss of the epithelium, thickening of certain areas, desquamation of epithelial cells, keratinization of epithelial cells, edema of epithelial tissue, presence of pigmented cells and goblet cells, presence of rete ridges in the epithelium, and the smoothness of the subepithelial basal layer. The stromal scores included regularity, density, presence of inflammation, presence of stromal edema, microvessel density in five 400X fields, and appearance of microvessels in subepithelial or deeper in stroma or subendothelial. The endothelial scores measure the presence of endothelium and the intactness of Descemet’s membrane. Curcumin NPs in hyaluronic acid hydrogel treatment showed significantly better epithelial regeneration than untreated rabbits and those treated with N-acetylcysteine, a standard treatment for ulcerative keratitis (*p*<0.001). The Curcumin NPs into Hyaluronic acid-based hydrogel and the Hyaluronic acid-treated groups showed significant differences in epithelial scores compared to those without treatment (*p* < 0.05 and *p* < 0.01, respectively). The stromal scores of the group treated with Curcumin NPs into Hyaluronic acid-based hydrogel-treated and Curcumin NPs showed significant differences compared to the untreated groups. (*p* < 0.01 and *p* < 0.05, respectively.) Despite using different treatments, there were no discernible differences in endothelial layer healing among the groups. (*p* > 0.05). Due to rapid corneal healing during the first 5–7 days, followed by a slower epithelialization phase, we enucleated the eyes on day 8^65^. Alkali exposure results in severe corneal edema and opacity accompanied by epithelial defects and irregularities^12^. Curcumin nanoparticles in Hyaluronic acid hydrogel treatment resulted in much-reduced edema compared to the untreated group. Previous studies have demonstrated that Curcumin NPs inhibit neovascularization. However, our study provides a more comprehensive analysis of the structural impact of Curcumin NPs^66,67^. Figure 7 illustrates the pathological results obtained from H&E staining.

### Cytokine measurement results

**Fig. 8:**
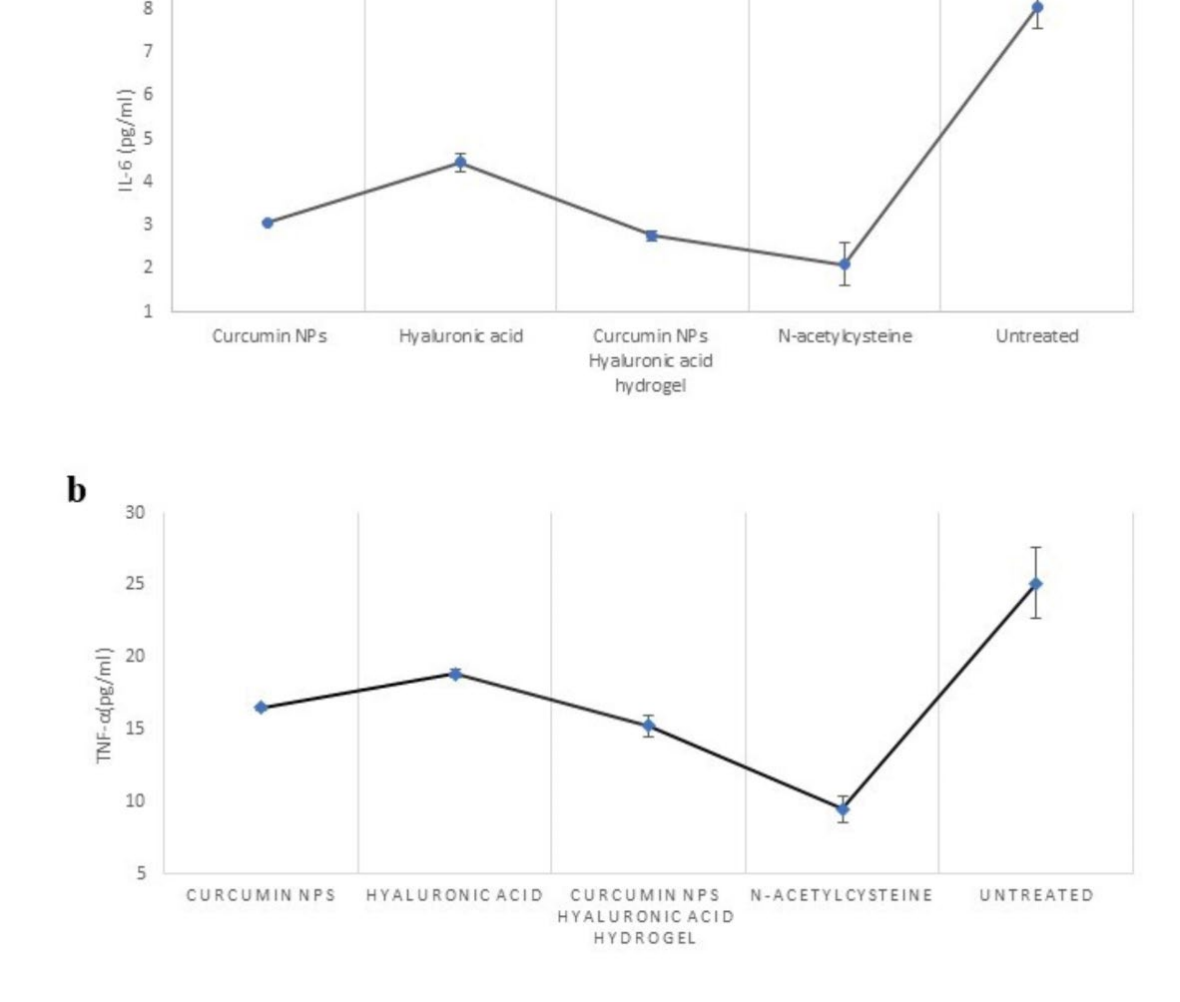
(**a**) IL-6 measurement in aqueous humour (pg/ml) (**b**) TNF-α measurement in aqueous humour (pg/ml).

We measured the levels of TNF-α and IL-6 in the aqueous humor as they may play a role in causing corneal melting. IL-6 and TNF-α, the primary molecular contributors to chronic inflammation, are the blockers of targets in the therapy of various inflammatory disorders^17^. After injury, there was an increase in the expression of proinflammatory cytokines in the cornea^68^. IL-6 is an acute inflammatory cytokine associated with pain, stimulates angiogenesis, and is a specific signal in keratitis^11,69,70^. In the treated groups, cytokine expression was downregulated, decreasing the clinical signs of inflammation, such as blepharospasm. Notable differences were observed compared to the untreated group based on the measurement results as the results are depicted in Fig. 8. (*p* < 0.05). The Curcumin NPs into Hyaluronic acid-based hydrogel treatment and the NAC treatment group had the least amount of these cytokines that showed the improvement of inflammation. Earlier research has shown that Curcumin NPs can reduce TNF-α and IL-6 levels in corneal and skin wound healing^67–69^, and HA hydrogel reduces IL-6 and TNFα in skin wound healing and corneal inflammation^14,71,72^. Previous research has demonstrated that curcumin can reduce angiogenesis in pterygium, an inflammatory disease in the cornea, by decreasing the expression and secretion of IL-6^19^.

These results confirmed the strong anti-inflammatory effects of Curcumin NPs into Hyaluronic acid-based hydrogel in corneal wound healing pathologically.

N-acetylcysteine is a potent MMP inhibitor and anti-inflammatory drug commonly used in human and veterinary ophthalmology^47^. One drop of acetylcysteine, a 5% solution, is recommended for the affected eye three times a day for seven days^73^. This study evaluated the efficacy of Curcumin NPs in Hyaluronic acid-based hydrogel topical eye drops in treating ulcerative keratitis. In several tests, it was superior to N-acetylcysteine.

## Conclusions

Curcumin was efficiently encapsulated in the β-cyclodextrin in Hyaluronic acid hydrogel using a simple and environmentally friendly process. Zeta, FTIR, and electron microscopy studies confirmed the formation of the inclusion complexes. Overall, these findings suggest that the pharmacological effects of topical Curcumin NPs encapsulated with β-cyclodextrin into Hyaluronic acid-based hydrogel for corneal re-epithelialization and anti-inflammation subsequently ameliorate ulcerative keratitis. Future studies should explore the synergistic impact of epidermal growth factor (EGF) loaded or artificial enzymes in topical Curcumin NPs encapsulated with β-cyclodextrin in Hyaluronic acid hydrogel in corneal wound healing. The therapeutic impact of these treatments, such as in alkali burn models, should be evaluated in different animal models. However, after ulcerative keratitis formation, there are many factors involved in corneal inflammation and neovascularization, and the mechanism is complex; therefore, further mechanistic research and drug experiments are still needed, such as determining the antioxidant activity and permeation medicine in surrounding ocular tissues, including the cornea, iris, lens, and conjunctiva. Our work opens new horizons for understanding the relationship between the coordination of curcumin NPs and Hyaluronic acid hydrogel, thereby providing a new potential strategy for treating ulcerative keratitis.

## Electronic supplementary material

Below is the link to the electronic supplementary material.

Supplementary Material 1

## Data Availability

The data that support the findings of this study are openly available in an Excel file in the supplementary data.

## Abbreviations

BID: Twice a day
TID: Three times a day
NP: Nanoparticle
